# Applicability of a short/rapid ^13^C-urea breath test for *Helicobacter pylori*: retrospective multicenter chart review study

**DOI:** 10.1186/1471-230X-12-8

**Published:** 2012-01-19

**Authors:** Hemda Schmilovitz-Weiss, Vered Sehayek-Shabat, Rami Eliakim, Eitan Skapa, Yona Avni, Haim Shirin

**Affiliations:** 1 Gastroenterology Unit, Hasharon Hospital, Rabin Medical Center, Petach Tikva, Tel-Aviv University, Tel Aviv, Israel; 2 Department of Gastroenterology, Rambam Medical Center, Technion, Haifa, Israel; 3 Gastroenterology Unit, Assaf Harofeh Medical Center, Tel Aviv, Tel-Aviv University, Tel Aviv, Israel; 4 Gastroenterology Unit, Wolfson Medical Center, Holon, Tel-Aviv University, Tel Aviv, Israel

**Keywords:** BreathID^®^, breath test, *Helicobacter pylori*, test duration

## Abstract

**Background:**

Carbon labeled urea breath tests usually entail a two point sampling with a 20 to 30-minute gap. Our aim was to evaluate the duration of time needed for diagnosing *Helicobacter pylori *by the BreathID^® ^System.

**Methods:**

This is a retrospective multicenter chart review study. Test location, date, delta over baseline, and duration of the entire test were recorded. Consecutively ^13^C urea breath tests results were extracted from the files over a nine year period.

**Results:**

Of the 12,791 tests results, 35.1% were positively diagnosed and only 0.1% were inconclusive. A statistically significant difference in prevalence among the countries was found: Germany showing the lowest, 13.3%, and Israel the highest, 44.1%. Significant differences were found in time to diagnosis: a positive diagnosis had the shortest and an inconclusive result had the longest. Overall test duration averaged 15.1 minutes in Germany versus approximately 13 minutes in other countries. Diagnosis was achieved after approximately 9 minutes in Israel, Italy and Switzerland, but after 10 on average in the others. The mean delta over baseline value for a negative diagnosis was 1.03 ± 0.86, (range, 0.9 - 5), versus 20.2 ± 18.9, (range, 5.1 - 159.4) for a positive one.

**Conclusions:**

The BreathID^® ^System used in diagnosing *Helicobacter pylori *can safely shorten test duration on average of 10-13 minutes without any loss of sensitivity or specificity and with no test lasting more than 21 minutes.

## Background

Among the non-endoscopic procedures used in diagnosing *Helicobacter pylori *(*H. pylori*), serology remains the most accepted. It is a widely available, inexpensive test with a high negative predictive value. However, the variable specificity, especially if the prevalence of *H. pylori *is low, and its relatively poor positive predictive value, limit the use of the test. Carbon labeled urea breath tests (UBT), which have a high sensitivity and specificity, are commonly used as a noninvasive method in detecting an active *Helicobacter pylori *(*H. pylori*) infection. UBT are the preferred method used in epidemiological studies, screening dyspeptic patients and assessing eradication or recurrence of the infection [1]. These tests usually entail a two point sampling with a 20 to 30-minute gap, and necessitate a mass spectrometry for analysis. Upon modifying the sampling method, immediate results can be achieved.

One such option includes real time continuous sampling ^13^C molecular correlation spectroscopy (MCS™) technology. The BreathID^® ^device (Exalenz, Israel) has been validated and cleared by the FDA and has also been previously used on children and adults [2,3]. This office-based system offers several advantages over the conventional mass spectrometry-based UBT, including an immediate test result, a standardized test drink (citric acid) and, most importantly for children, a sampling method that does not require active cooperation.

For many years, test shortening procedures have been routinely practiced including the use of various ^13^C-urea concentrations, and different citric acid test drinks. The new molecular correlation spectrometry technology enables continuous sampling of the expired breath of the patients, which in turn, enables the device to terminate the test immediately after a conclusive positive or negative result of *H. pylori *had been identified [2].

The use of a citric acid-based test drink has been shown to enhance hydrolysis of the urea and produce a more rapid rise in expired ^13^CO_2 _[4]. The BreathID™system combined with a citric acid drink and continuous breath sampling significantly reduces the amount of time needed for a final result compared to isotope ratio mass spectrometer. However, these test-shortening modifications have not been widely accepted by the medical community. There are limited data [5] validating these tests but they have been in use in several gastroenterology departments for many years.

The current investigation retrospectively examined over 12,000 BreathID™ test results from several randomly selected gastroenterology departments. The goal of this retrospective evaluation was to assess, on average, the minimal time required to achieve accurate, definite UBT results using the BreathID™ system.

## Methods

### Urea Breath Tests

The effect of the breath test sampling method in detecting *H. pylori *was examined using continuous real time methodology (i.e., the BreathID^® ^system). The BreathID™ system is comprised of the following components: a) a kit containing 75 mg of ^13^C-urea (a 99% ^13^C-enriched urea tablet); b) a packet of granulated Citrica (a 4.5 gram packet containing 4 g of citric acid, 0.149 mg of aspartame, orange aroma, FD&C yellow #6); c) an IDcircuit-sampling device; and d) a BreathID^® ^device. All patients received 75 mg of ^13^C-urea with a 4.5 gram citric acid based powder (Citrica). The IDcircuit, a continuous nasal breath sampling device, transported the breath sample from the patient to the BreathID™ and did not require active cooperation.

Based on molecular correlation spectrometry, the BreathID^® ^continuously measured ^13^CO_2 _and ^12^CO_2 _concentrations from the patient's breath, thus establishing the ^13^CO_2_/^12^CO_2 _ratio, displayed vs. time on the screen. The results were obtained within 10-15 minutes and printed on a thermal printer.

Determining the positive or negative results from the BreathID™ was based on a device algorithm. If, after 5 minutes, the delta over baseline (DOB) of the ^13^CO_2_/^12^CO_2 _ratio was greater than 6 at more than two time points, the patient was considered positive. If, after 5 minutes, the DOB was below 3 at more than two time points, the patient was considered negative. If, after 20 minutes, neither of the two criteria was fulfilled, then the nominal 5 DOB threshold was considered in order to distinguish positive from negative patients, unless the previous three points were within ± 1 DOB of the 5 DOB threshold. In this case, the results were defined as inconclusive.

The study protocol was approved by the Institutional Review Board of the Rabin Medical Center. Informed consent was not taken from all patients since this is retrospective study with data taken from different centers not all needing informed consent. The study was supported by Exalenz, Ltd.

### Study Subjects and Protocol

A total of 12,751 consecutively selected ^13^C urea breath tests performed between 2001 and 2009 were extracted from the files of fifteen gastrointestinal units in Israel, The Netherlands, Switzerland, Germany and Italy, representing approximately 50% of the tests performed during the 9-year period. Some patients underwent a second UBT test after H. *pylori *eradication. For subjects who had more than one test result, pre and post treatment, all of the identified tests were used. There was no obvious selection bias, as all tests were collected from these centers. ^13^C-UBT in the gastroenterology clinical laboratories was used in subjects with gastrointestinal symptoms, such as dyspepsia, peptic ulcers and gastric malignancy. UBT was given to patients after a three-hour fast.

Exclusion criteria included: a) administration of antibiotics and/or bismuth preparations within four weeks prior to date of entry to the study; b) administration of proton pump inhibitors within 2 weeks prior to date of entry to the study; and c) pregnant or breast-feeding women. The challenges were separated into positive (DOB > 5 within 20 minutes) and negative test results (DOB < 5 within 20 minutes). Test date, country, device number, DOB, final result time (time at which the test result was obtained) and duration of the entire test were recorded. In addition, we specifically searched for the number of individuals who had an inconclusive result after 20 minutes (within 1 DOB of the threshold, as described above).

### Statistical Analyses

Continuous variables are summarized by mean, standard deviation (SD), minimum, median and maximum and are compared by a t-test (two groups) or ANOVA (3 groups or more). Categorical variables are summarized by a count and percentage and compared by a chi-squared test. Since so few tests were inconclusive, subjects with inconclusive results were excluded from the analyses. In addition, there were 27 test subjects for whom duration of examination was incorrectly recorded due to a software bug. Therefore, these cases were also excluded from analyses of test duration.

## Results

### Prevalence of *H. pylori*

The diagnosis distribution was evaluated in all sites within each country. Italy - 7, Israel - 5 and Germany, The Netherlands and Switzerland, with only one site each. All sites had one device per site except for Switzerland, with two.

Study data are presented in Table 1. Out of 12,751 tests performed (Table 1), only 0.1% of the test results were inconclusive. Analysis of the *H. pylori *prevalence by country revealed a statistically significant difference in prevalence among the countries evaluated in the study. Germany had the lowest prevalence with 13.3% and Israel the highest with 44.1% (Table 2).

**Table 1:** Distribution of test duration and DOB by diagnosis.

Outcome	N (%)	Distribution	by test	duration	(n = 12,724)*
		
		Mean ± SD (Minutes)	Minimum (Minutes)	Median (Minutes)	Maximum (Minutes)
**Inconclusive**	8 (0.1)	18.04 ± 1.76	14.68	18.09	20.35
**Negative**	8254 (64.8)	10.03 ± 2.24	5.43	10.05	20.58
**Positive**	4462 (35.1)	8.93 ± 2.55	5.42	8.02	20.90
**Outcome**	N (%)	**Distribution**	**of DOB by**	**diagnosis**	**(n = 12751)**
		**Mean ± SD**	**Minimum**	**Median**	**Maximum**
**Inconclusive**	8 (0.1)	5.50 ± 0.24	5.20	5.55	5.80
**Negative**	8268 (64.8)	1.03 ± 0.86	-0.90	1.00	5.00
**Positive**	4475 (35.1)	20.20 ± 18.89	5.10	13.20	159.4

*(There were 27 tests without duration results, only DOB results (as mentioned in the paper).

**Table 2:** *H. pylori *prevalence by country

	Diagnosis				
		
Country	Negative		Positive		All
	N	%	N	%	
Germany	169	86.7	26	13.3	195
Netherland	1671	76.0	529	24.0	2200
Israel	3322	55.9	2619	44.1	5941
Italy	2701	70.1	1154	29.9	3855
Switzerland	405	73.4	147	26.6	552
P value*	< 0.0001				

*Between countries

### Duration of the Testing Procedure

The duration of the test procedure from initiation until completion (overall test duration) and the amount of time required until the device was able to provide a diagnosis (test duration until diagnosis) was compared. We found significant differences in both overall duration and time to diagnosis obtained among the three possible outcomes: positive, negative and inconclusive: a positive diagnosis had the shortest duration and an inconclusive result had the longest duration (Table 1). Significant differences in both overall duration and time to diagnosis between the countries were also found.

Overall, test duration was 15.1 minutes in Germany versus approximately 13 minutes in other countries. This may have been due to the low prevalence of *H. pylori *in Germany (Table 2) which lead to a longer test duration (Table 3). Diagnosis was obtained after approximately 9 minutes in Israel, Italy and Switzerland, but after 10 minutes in The Netherlands and Germany. Significant differences in both overall duration and time to diagnosis among the sites were also demonstrated, where the shortest mean overall test duration was 12.7 minutes in Italy versus approximately 15 minutes in Israel and Germany. Diagnosis was obtained after approximately 9 minutes in three Israeli sites, one Italian site and a little over 10 minutes on average in The Netherlands, Germany and two Italian sites. Similar results were obtained between devices within their respective countries, which may validate the differences as country- related and not device- related.

**Table 3:** Distribution by country of total test duration and test duration until diagnosis

				Test duration (min)						Test duration until diagnosis (min)		
Test duration by country	N	Mean	SD	Minimum	Median	Maximum	N	Mean	SD	Minimum	Median	Maximum
**Country**												
**Germany**	195	15.10	4.18	10.43	14.48	32.77	195	10.24	2.43	6.68	10.25	20.18
**Netherland**	2198	13.36	2.97	6.63	12.95	41.40	2198	10.46	2.58	5.42	10.26	20.48
**Israel**	5918	13.13	3.18	8.18	12.05	47.12	5918	9.23	2.20	5.62	8.25	20.48
**Italy**	3853	12.97	3.25	8.17	11.68	55.37	3853	9.78	2.47	5.62	8.65	20.90
**Switzerland**	552	13.80	4.01	8.53	12.94	33.28	552	9.59	2.47	6.12	8.83	20.38
**P value**	< 0.0001						< 0.0001					

No significant differences were found in overall test duration between tests performed in the morning (until 4 pm) versus tests performed in the afternoon/evening (4 pm or later).

### Delta Over Baseline Values (DOB)

Diagnosis, given by the BreathID™ device, was derived by evaluating the DOB value. The mean DOB value for a negative diagnosis was 1.03 (± 0.86) with a range of 0.9 to 5, whereas the positive cases had a mean DOB of 20.2 (± 18.9) and a range of 5.1 through 159.4. Greater variability was seen in DOB means between countries for positively diagnosed subjects than for negative subjects, although this difference was not statistically significant (Table 1).

## Discussion

The optimal ^13^C-UBT conditions for diagnosing *H. pylori *infection are still being perfected. Attempts to improve the ^13^C-UBT have focused on decreasing the amount of substrate used and reducing the duration of the test. Urita et al. recently reported an ultra short ten second endoscopic UBT using 20 mg of ^13^C-urea sprayed onto the gastric mucosa. The maximum sensitivity and specificity of intragastric samples were 83.7% and 100% with a cut-off point of 8 per thousand. However, their clinical cohort cannot be compared with ours because of the use of an invasive endoscopic method [6].

No consensus was demonstrated in the evaluation of different ^13^C-UBT protocols regarding the dosage of the ^13^C-urea, the time and interval of the breath sample collection or the test meal chosen to delay gastric emptying. Each clinical center used its own test protocol, making comparison of results almost impossible. Although Dominguez-Munoz et al. reported identical sensitivity and 100% specificity of ^13^C-UBT for three different test meals (0.1 N citric acid solution, semiliquid fatty meal and semiliquid meal), the delta peak values of ^13^CO_2 _were much higher when a citric acid solution was used as the test drink [7]. Moreover, Graham et al. using 1, 2 and 4 g of citric acid reported that the increase in urease activity was dose dependent [8].

In general, acidic gastric milieu may improve the accuracy of the UBT, probably by increasing the entrance of urea into *H. pylori *and the activity of its cytoplasmic urease, the cornerstone of the urea breath test [9-12]. Acidic gastric juices may also neutralize the ammonia, which may cause bacterial damage by itself and reduce urease activity [13].

A higher acidic gastric environment (pH approximately 2.0) induced by citric acid has been found by several investigators to increase the exhaled CO_2 _isotopes (^13^C or ^14^C) levels in a dose dependent manner [4,7,8]. To maintain a low gastric pH, we used high dose citric acid, which also delays gastric emptying [14]. However, others have hypothesized that these two factors appear unlikely to be the critical determinants in the increased access of urea to the urease enzyme *in vivo *[15-17].

Hamlet et al. more than a decade ago, examined the efficacy and duration of ^14^C-UBT using similar methods [18,19]. They reported that by supplying ^14^C-urea as a rapid -release tablet along with citric acid, it is possible to shorten the duration of the UBT to 10 minutes with excellent accuracy, even during acid suppression therapy. The tablet-based UBT proved to be accurate during omeprazole treatment, correctly identifying all of the 10 *H. pylori*-infected patients [19].

Chey et al. and our group also demonstrated that intragastric acidification by citrate administration before and during the UBT decreased the false negative results in patients receiving PPI treatment [20-22]. The decreased false negative results induced by PPIs are probably related to the use of citric acid as a test drink. Thus, the data reported by Hamlet et al. are consistent with our data.

An acknowledged weakness of our study was the absence of a gold standard to evaluate the performance of the BreathID^® ^system. However, the device was approved by the FDA in 2001, making it a comparable test to endoscopy in diagnosing *H. pylori*. (The validation trial can be reviewed at link: http://www.accessdata.fda.gov/cdrh_docs/pdf/k011668.pdf).

Since our data is based on raw data from the devices, demographic data is unavailable. The impact of this office- based, fully automated breath collection system, showing immediate results, is that it is activated by a single button, with no need of entering other patient data. Nevertheless, the patient's name/ID can be added to the printout.

## Conclusions

The BreathID^® ^test used in our research, performed with a continuous breath test sampling device, collected data from 5 countries over a period of 9 years. Our analysis of 12,791 randomly chosen BreathID™ tests using a high dose citric acid as a test drink and continuous sampling of the expired breath indicated that completion of the UBT required 10-13 minutes on average. Only 8 subjects (0.1%) from the total population had inconclusive results and needed further time to reach a conclusive result. Our results are in agreement with an earlier report using the same technique on a smaller group of examinees [5]. Although this study is retrospective and *post hoc *in design, our results provide validation of the usefulness of BreathID^® ^as a short and rapid method in diagnosing *H. pylori*.

## Competing interests

The authors declare that they have no competing interests.

## Authors' contributions

HS-W participated in the design of the study, drafted the manuscript and conceived the study. VS-S collected, analyzed and interpreted the data. RE collected, analyzed and interpreted the data. ES collected, analyzed and interpreted the data. YA collected, analyzed and interpreted the data. HS participated in the design of the study, drafted the manuscript and conceived the study.

All authors read and approved the final manuscript.

## Pre-publication history

The pre-publication history for this paper can be accessed here:

http://www.biomedcentral.com/1471-230X/12/8/prepub
